# Zika Virus and Host Interactions: From the Bench to the Bedside and Beyond

**DOI:** 10.3390/cells9112463

**Published:** 2020-11-12

**Authors:** Daniel Limonta, Tom C. Hobman

**Affiliations:** 1 Department of Cell Biology, University of Alberta, Edmonton, AB T6G 2H7, Canada; tom.hobman@ualberta.ca; 2 Li Ka Shing Institute of Virology, University of Alberta, Edmonton, AB T6G 2E1, Canada; 3 Department of Medical Microbiology & Immunology, University of Alberta, Edmonton, AB T6G 2E1, Canada; 4 Women & Children’s Health Research Institute, University of Alberta, Edmonton, AB T6G 1C9, Canada

**Keywords:** zika, host, interferon, cell death, placenta, NS5, peroxisome, mosquito, guinea pig, tight junction

Before the emergence of SARS-CoV-2 (severe acute respiratory syndrome coronavirus 2), the causative agent of the current COVID-19 (coronavirus disease 2019) pandemic [1], another RNA virus, Zika virus (ZIKV) belonging to the flavivirus family, re-emerged and was declared a Public Health Emergency of International Concern by the WHO in February 2016 because of clusters of microcephaly cases and other neurological disorders in Brazil [2]. Until that time, ZIKV was known as a cause of a self-limited febrile disease similar to dengue fever and other arboviral febrile diseases that occur in tropical and subtropical regions [3]. The scientific community was surprised to find that a mosquito-borne flavivirus could also be transmitted to fetuses during pregnancy and to sexual partners. So far, no vaccines or antiviral drugs are licensed for ZIKV and the virus has spread and become endemic to many Latin-American countries. Furthermore, because of increased international travel and trade, this virus has also spread to some developed nations [4].

In this Special Issue in *Cells*, Zika virus and host interactions, thirteen articles that address diverse aspects of ZIKV–host interactions from basic science to clinical research are presented (Figure 1). Arboviral diseases are infectious diseases in which a third key element beside the pathogen and susceptible individuals is involved—arthropods. In this Special Issue, Martinez-Rojas et al. [5] showed how extracellular vesicles (EVs) released from ZIKV-infected mosquito cells (C6/36) may affect not only bystander mosquito cells, but also human cell types, that are important ZIKV targets. Isolated EVs can infect and promote a pro-inflammatory state in human monocytes and microvascular endothelial cells. These modifications of cellular behavior may contribute to ZIKV transmission and pathogenesis in vector and human host cells.

Animals models are critical for understanding viral pathogenesis and testing therapeutic interventions. To this end, Saver et al. [6] inoculated guinea pigs by subcutaneous and vaginal routes to mimic the infected mosquito bite and sexual transmission, respectively. By studying multiple tissues with respect to tropism and persistence, they identified sensory and autonomic ganglia in the nervous system as a potential novel niche of ZIKV persistence.

The unusual pathogenic link of ZIKV with the male reproductive system was reviewed by Almeida et al. [7]. The infection and persistence of ZIKV in the testes leading to sexual transmission may impact reproduction by a number of physiological and immunological mechanisms. During pregnancy, infection of the placenta after sex with the infected partner can lead to fetal infection and the development of congenital Zika syndrome [4].

Also included in this Special Issue are two reports focused on interactions of ZIKV with placental cells. Miranda et al. [8] showed that syncytiotrophoblast cells of placentae from ZIKV-infected women display increased paracellular permeability in comparison with placentae from non-infected women, a phenomenon that may be linked to distribution of tight junction proteins. Results from transepithelial electrical resistance assays using a human trophoblast cell line supported the in vivo findings. Rabelo et al. [9] analyzed another cell type from the placenta, the mast cells. These authors detected ZIKV NS1-positive mast cells in the placenta of infected women and then using a human mast cell line permissive for ZIKV infection, looked at virus-induced ultrastructural changes. Furthermore, degranulation kinetics and production of pro-inflammatory cytokines and vascular endothelial growth factor were triggered shortly after ZIKV infection of these cells. The authors suggest that mast cells may contribute to associated inflammation and viral spread in placenta.

A productive infection of host cells by ZIKV would not be possible without effective evasion of the innate immune response. Several articles in this Special Issue addressed different mechanisms used by ZIKV to overcome the antiviral response of the host. Apoptosis is effective in clearing viruses from tissues and any delay of this process may facilitate viral production and spread. Turpin et al. [10] demonstrated that ZIKV replication inhibits intrinsically and extrinsically induced apoptosis in A549 cells. They also showed that control of apoptosis during viral infection may be mediated by the anti-apoptotic bcl-2 family protein. Schilling et al. [11] used CRISPR/Cas9-mediated knockouts and transcriptomic analysis in A549 cells to show that RIG-I was the main sensor required to induce a protective type I IFN response during ZIKV infection. Surprisingly, RIG-I-mediated signaling also prevented A549 cells from viral-induced apoptosis. Specifically, loss of RIG-I led to apoptotic activation 4–6 days after infection, which may be related to reduced type I IFN signaling and increased virus multiplication in RIG-I-deficient cells. Furthermore, these authors showed that ZIKV non-structural protein NS5 not only suppresses IFNAR signaling, but also inhibits transcription of type I interferon genes.

The critical role of the flaviviral NS5 in evading the antiviral response against ZIKV was further explored by two other groups. Roby et al. [12] showed that NS5 of ZIKV as well as those from other flaviviruses, such as West Nile virus and Japanese encephalitis virus, antagonize host cell Janus kinase/signal transducer and activator of transcription (JAK/STAT) signaling downstream of interferons α/β. This is caused in part by NS5 binding to cellular heat shock protein 90 (HSP90) in replication complexes, resulting in suppression of JAK/STAT-dependent cytokine signaling. NS5 binding not only destabilized HSP90, but also disrupted the interaction of this chaperone with other client kinases. In a review article, Tan et al. [13] discussed how nuclear localization of ZIKV NS5 in placental and neural cells antagonizes type I interferon signaling and contributes to the transcriptional activation of pro-inflammatory genes. These findings support the possible role of ZIKV NS5 nuclear spherical-shell structures in the modulation of the host immune response.

The six other non-structural proteins encoded by ZIKV also interact with a number of human host proteins. Golubeva et al. [14] generated an interaction network built on previous interaction networks of ZIKV and a combination of tandem-affinity purification coupled to mass spectrometry with yeast two-hybrid screens to identify 150 human proteins interacting with ZIKV non-structural proteins. In validation experiments, they showed that the protein inhibitor of activated STAT1 (PIAS1) binds to NS5 and modulates its stability. Furthermore, this interactome study validated proteins known to be associated with microcephaly disorders.

Wong et al. [15] addressed how peroxisomes, which are metabolic organelles with key roles in antiviral defense, are affected by ZIKV infection. Peroxisomes function as signaling platforms for the interferon response, and dysregulation of peroxisome biogenesis can alter antiviral signaling. The authors demonstrated that ZIKV infection depletes peroxisomes in human fetal brain cells, while the peroxisome biogenesis factor PEX11B restricts ZIKV replication likely by increasing peroxisome numbers and enhancing downstream antiviral interferon signaling. ZIKV capsid protein was shown to interact with the peroxisome biogenesis factor PEX19, resulting in its degradation and causing loss of peroxisomes. As peroxisome function and numbers can be pharmacologically modulated, this study suggests that further investigation of peroxisome-based antiviral strategies for ZIKV and other viral infections is warranted.

Bos et al. [16] showed the importance of amino acid residues isoleucine 152 (Ile152), threonine 156 (Thr156), and histidine 158 (His158) (E-152/156/158) in the ZIKV envelope (E) protein for infection of host cells. Of note, these three amino acid residues surround an important *N*-glycosylation site (E-154) in E protein that is associated with ZIKV infectivity. To illustrate the importance of these E protein residues in ZIKV infection, the authors swapped them between the Brazilian epidemic strain BR15 and placed them with the corresponding amino acids from the pre-epidemic African strain MR766. The BR15 virus containing the E-152/156/158 residues from MR766 was less infectious in vitro than the parental virus. In contrast, the MR766 virus containing amino acid residues 152/156/158 from the Brazilian strain was more infectious. As changes to this region of E protein did not affect virus binding to host cells, virus-induced cell death, or the interferon response, the authors speculated that they may be important for infectivity by promoting viral membrane fusion inside infected cells. These findings may contribute to the design of innovative strategies to control ZIKV infection at early stages.

Finally, regarding T-cell immunity to ZIKV, the pre-existing immunity to the related flavivirus, dengue virus (DENV), has been discussed as a risk factor for immunopathological responses [4]. Pardy and Richer [17] reviewed current evidence from epidemiological studies and different mouse models about CD4 and CD8 T-cell responses to ZIKV. They concluded that the issue of whether prior DENV immunity provides cross-protection to ZIKV remains an open question that should be further investigated. Furthermore, a better understanding of T-cell responses to ZIKV and the mechanism by which this virus evades cell-mediated immunity will be important for designing effective anti-ZIKV vaccine candidates.

We hope that this Special Issue will be of wide interest, particularly to those researchers focused on different aspects of ZIKV–host interactions. It is expected that findings from these studies will contribute to a better understanding of the vector and human host cells interacting with ZIKV, and will serve as the basis for novel diagnostics, antiviral therapeutics, and vaccines.

## Figures and Tables

**Figure 1:**
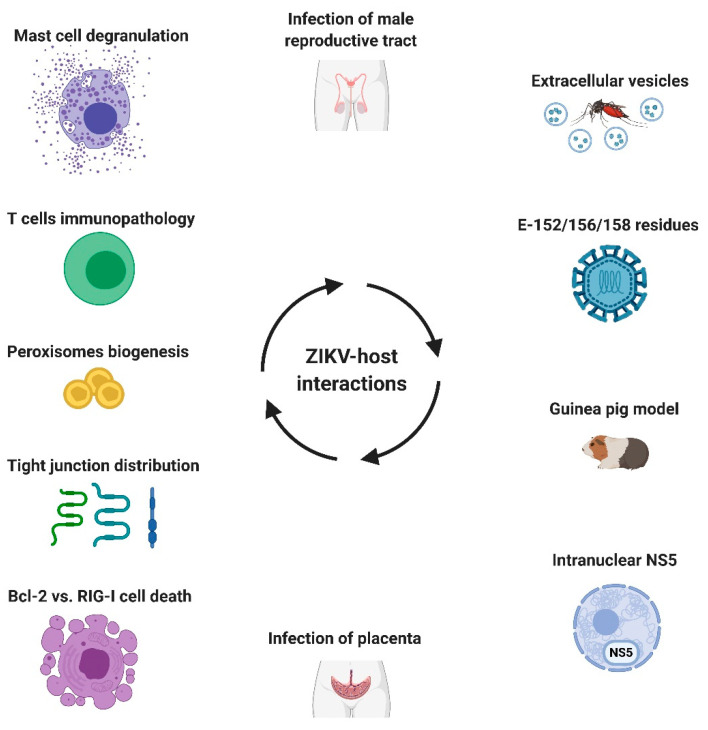
Zika virus (ZIKV)–host interactions. ZIKV and host interactions were addressed in this Special Issue by using in vitro, ex vivo, and animal models together with clinical samples. The complex interplay between host factors and viral components in different cell types and tissues dictates the outcomes of infection: aborted or productive infection, clinical presentation, and disease severity. Created with BioRender.com.
